# Hematological Indices in Children with Non-organic Failure to Thrive: a Case-Control Study

**Published:** 2016-03-15

**Authors:** AH Jafari Nodoshan, A Hashemi, A Golzar, F Karami, R Akhondzaraini

**Affiliations:** 1 **Department of Pediatrics, Shahid Sadoughi University of Medical Sciences, Yazd, Iran.**; 2 **Hematology and Oncology Research Center, Shahid Sadoughi University of Medical Sciences and Health Services, Yazd, Iran. **; 3 **Children Growth Disorder Research Center, Shahid Sadoughi University of Medical Sciences, Yazd, Iran.**; 4 **Shahid Sadoughi University of Medical Sciences, Yazd, Iran. **; 5 **Infectious Diseases Research Center, Shahid Sadoughi University of Medical Sciences, Yazd, Iran.**

**Keywords:** Ferritin, Hematological indices, Non-Organic Failure to Thrive

## Abstract

**Background:**

Non-organic failure to thrive (NFTT) is the most common cause of failure to thrive (FTT) which is attributed to inadequate nutrition due to economic factors or parental neglect***. ***NFTT can lead to a vicious cycle of poor and inadequate eating and severity of anemia. The aim of this study was to determine the hematological indices in children with NFTT*.*

**Materials and Methods:**

In a cross sectional case control study, iron status and blood indices of forty five aged 6–60 months children with NFTT were evaluated and compared with 45 healthy control children (with matching of age and sex).

**Results:**

In this study, the prevalence of anemia was 48.9% in NFTT compared to 11.4% in the control group (p<0.001). Microcytic anemia was significantly more prevalent among the subjects than the controls (77.8% versus 27.3%; p<0. 001). The serum iron level was 73.2 and 62.8 mcg/dl for the case and control groups (P=0.29). The ferritin level in the study group was 29.8 versus 35.47 ng/ml in the control group (p=0.227). The prevalence of iron deficiency anemia among children with mild, moderate, and severe underweight was 44.4%, 45.5%, and 48%, respectively. The highest prevalence of iron-deficiency anemia was seen between age group of 12 and 24 months (p<0.05).

**Conclusion:**

Based on the results of this study, a correlation between malnutrition and anemia was found. However, further studies are needed to assess and confirm the current outcomes.

## Introduction

Failure to thrive and anemia are the most serious health problems not only in developing countries, but also throughout in the world (1). According to a 1998 nationwide study, 10.9% of children aged less than five years were underweight (2). The most common causes for failure to thrive are inadequate food intake, low socioeconomic status, and low educational level. The measurement of iron status has been recognized as a strong objective and quantitative data on nutritional status. Iron deficiency (ID) is the most common micronutrient deficiency the world over, affecting a large number of persons in each country. Iron deficiency is the most common cause of anemia. There are many different causes and types of anemia that vary not only from country to country, but also within regions and different ethnic populations within countries. The World Health Organization (WHO) reported that more than two billion people worldwide are anemic (3). According to World Health Organization classification, the prevalence of anemia in Iran is in the moderate category, which means that about 18% to 38% of Iranian younger than 5 years is anemic (4). Iron requirements are particularly high during periods of rapid growth, such as childhood (5) resulting in stages of high risk for ID. Appetite in infants with abnormal growth is more affected by iron deficiency anemia, aggravating the anemia in such situations. So, the presence of anemia with normal growth is not common and further investigations are needed. A large portion of iron deficiency is preventable with appropriate and timely intervention. However, the relationship between failure to thrive and iron deficiency should be better understood so that appropriate measures might be taken to prevent these problems. 

## Materials and Methods:

This study carried out among children, aged less than five years old between May 2013 and August 2014 in primary health care centers of Taft city, Iran. The case group consisted of 45 children aged between 6 and 60 months who admitted children with non-organic failure to thrive. Non-organic failure to thrive has been defined as a child whose weight (or weight for height) was more than 2 SD below the mean for sex and age, and/or child whose weight curve crossed downward more than 2 major percentile lines. The control group consisted of healthy age and sex matched control children that were referred for routine health care to a primary health care center. The exclusion criteria consisted of receiving an iron combination within the past month and the presence of any chronic systemic diseases (cardiac, renal, metabolic among others). The Gomez classification of nutritional status was used to classify malnutrition in children, based on percentage of expected weight for age: over 90% is normal, 76-90% is mild (first degree) malnutrition, 61-75% is moderate (second degree) malnutrition and less than 60% is severe (third degree) malnutrition. To determine the hematological parameters, blood samples were collected from all patients and analyzed for: serum ferritin level, serum iron level, mean corpuscular volume (MCV), mean corpuscular hemoglobin (MCH), hemoglobin (Hb), and haematocrit (Hct). Anemia is defined as hemoglobin level of less than 11 g/dl, Microcytic anemia is defined MCV<75 μm3 or fl and Iron deficiency is defined as serum ferritin level < 12 ng/ml. Informed consent was taken from the parents and the study was approved by the Ethics Committee of Shahid Sadoughi University of Medical Sciences, Yazd, Iran.

**Statistical Analysis**

The data was analyzed using SPSS 17.0. Chi-square test was used for data analysis of qualitative variables, and mean values were compared using independent T-test. Differences were considered significant if at P-values < 0.05.

## Results

This study was done on 90 children including 45 boys and 37 girls with mean age of 3.32±1.27 year. Mean hemoglobin levels were significantly lower in underweight children as compared to those who were normal. Values of MCV and MCH were significantly lower (p < 0.001) in Non-organic failure to thrive (NFTT) children than in their healthy peers (Table І). When classified by the combination of serum ferritin, serum iron, and hemoglobin values, 48.9% of the underweight children were recognized as anemic (Hb concentration<11g/dl), 33.3% in a state of iron deficiency (serum iron<60μmol/L), and 5.6% with low iron stores (serum ferritin <12μg/L).There was no significant difference in the prevalence of iron deficiency between two groups (P=0.345). Using a cut-off value of 75 fl for MCV, 77.8% of the children with NFTT had microcytic anemia. Data on Serum ferritins were available for 72 (81%) of participants. No significant difference was found in the prevalence of low ferritin levels (P=0.151). The prevalence of anemia and iron status indicators among children are is shown in Table II**.** According to the results, 55.6% of the patients had severe malnutrition, 24.4% suffered from moderate malnutrition and 20% have mild malnutrition at the time of examination. The relationships between severity of malnutrition and hematological indices are shown in Table III. There was no significant difference in the mean corpuscular hemoglobin concentration and iron status with regard to type of severe malnutrition. (P=0.426 and P=0.275, respectively). The prevalence of anemia was 44.4 %, 45.5%, and 48% for mild, moderate, and severe malnutrition, respectively (P=0.896) Table III. The prevalence of anemia was significantly different between age groups (P<0.001). The highest prevalence of iron-deficiency anemia was in 12- to 24-month age group (p<0.05) and Hb levels below the normal values (<11 g/dl) decreased with age )Table IV).

**Table I:** *Hematological indices of enrolled children (mean±SD*

Group	Hb (mg/dL)	MCV(fL)	MCH(pg)	Iron μmol/L	Ferritin μg/L
NFTT	11.02±1.3	71.2±5.8	25.3±2.7	73.2±24	29.8±17
Healthy	12.1±0.81	76.4±3.2	27.1±1.6	62.8±12.7	35.47±21
p-value	<0.001	<0.001	0.001	0.29	0.227

**Table II:** Iron status indicators, prevalence of anemia and IDA among children

Group	Hb< 11 (g/dL)	MCV< 75 (fL)	Ferritin <12 (μg/L)	Iron <60(μmol/L)
NFTT	48.9%	77.8%	5.6%	33.3%
Healthy	13.9%	33.3%	19.4%	44.4%
p-value	0.001	<0.001	0.151	0.345

**Table III:** *Hematological indices according to the type of malnutrition*

Degree	Hb< 11 (g/dL)	iron<60(μmol/L)	MCV< 75(fL)
**Mild**	44.5%	50.0%	83.3%
**Moderate**	45.5%	33.3%	88.9%
**Sever**	48.0%	0.0	60.0%
**p-value**	0.896	0.275	0.426

**Table IV:** *Prevalence of anemia* *between age groups*

**Age group**	**1-2**	**2-3**	**3-4**	**>4**	**p-value**
**≤11**	15	8	2	2	≤0.001
**>11**	5	17	15	17	

## Discussion

Failure to thrive (FTT) is common among preschool children and is usually coupled with anemia (6). This study demonstrated a high prevalence of anemia (48.9% vs. 11.4%) and Microcitic anemia (77.8 % vs 27.3%) among the children with FTT in comparison with control group. However, Frequency of iron deficiency was not significantly different between two groups (p>0.05). In previous study carried out by Hajishabani et al (7) the prevalence of anemia in healthy children (<5 years old) of Yazd reported as 20.8%. A national survey of 4170 children in Iran estimated that the prevalence of iron deficiency is around 16 to 21% in 2-6 years group (8). Although some studies have reported a high prevalence of childhood anemia in India (72.2%) (9) and Ghana (78.4%) (10). This difference may be due to numerous factors such as socio-economic status, iron intake, prevalence of parasitic and infectious diseases. The current findings highlighted and strengthened previous observations (11-13) that significant underweight is correlated with anemia. In Ilam, a survey by Ghiasi et al. (14) reported a high prevalence of anemia (67.8%) among malnourished children less than 5 years old. Other study in India also confirmed that very high percentage (67.3%) of malnourished children was found to be anemic (13). However in Nor Aini et al. (12) study in Malaysia, iron deficiency defined as serum ferritin level below10 ng/dl was more frequent in children with wasting and on the other hand, serum iron and hemoglobin levels were lower in underweight and stunting children. Similar observations have been also reported in children less than 5 years (15) and in school-aged children (16). The current findings were in contrast with Keikhaei et al (17) who reported no significant correlation between iron-deficiency anemia and anthropometric measurements. Molavi and Nazemi showed that Iron supplementation improves growth indexes in children (18). Several studies have explored the association between the micronutrient deficiency and growth failure (19). The decline in prevalence of IDA among the older children in this study supports findings in previous studies (10, 20). Another study in Ahwaz province showed that most children with IDA were at 12-23 months (21). The measurement of Hb concentration in whole blood is most widely used as a screening test for anemia. In this study, hemoglobin concentration and MCV levels were correlated with underweight so they can be better markers for the assessment of anemia. In contrast to these finding, Ghosh et al. revealed that the serum ferritin can be identified as a sensitive marker for the measurement of iron status in surveyed children (11). These findings were in contrast with other studies that reported the association of significant underweight and IDA (6, 14). 

## Conclusion

Anemia and iron deficiency anemia were more frequent in children with NFTT. Therefore, early diagnosis and treatment of anemia is essential in these patients. Further studies, on a larger population of children, to determine the role of other factors is needed.

## Conflict of interest

The Authors have no conflict of interest.
